# Structure and stability of an apo thermophilic esterase that hydrolyzes polyhydroxybutyrate

**DOI:** 10.1107/S2059798324009707

**Published:** 2024-10-23

**Authors:** Gwendell M. Thomas, Stephen Quirk, Raquel L. Lieberman

**Affiliations:** a School of Chemistry and Biochemistry Georgia Institute of Technology 901 Atlantic Drive NW Atlanta GA30332 USA; b Kimberly-Clark Corporation, 1400 Holcomb Bridge Road, Roswell, GA30076, USA; F. Hoffmann-La Roche Ltd, Switzerland

**Keywords:** serine hydrolases, bioplastic degradation, thermal stability, polyhydroxybutyrate

## Abstract

Pollution from plastics is a global problem that threatens the biosphere for a host of reasons, including the time scale that it takes for most plastics to degrade. Biodegradation is an ideal solution for remediating bioplastic waste as it does not require the high temperatures necessary for thermal degradation and does not introduce additional pollutants into the environment. Numerous organisms can scavenge for bioplastics, such as polylactic acid (PLA) or poly-(*R*)-hydroxybutyrate (PHB), which they can use as an energy source. Recently, a promiscuous PHBase from the thermophilic soil bacterium *Lihuaxuella thermophila* (*Lt*PHBase) was identified. *Lt*PHBase can accommodate many substrates, including PHB granules and films and PHB block copolymers, as well as the unrelated polymers polylactic acid (PLA) and polycaprolactone (PCL). *Lt*PHBase uses the expected Ser–His–Asp catalytic triad for hydrolysis at an optimal enzyme activity near 70°C. Here, the 1.75 Å resolution crystal structure of apo *Lt*PHBase is presented and its chemical stability is profiled. Knowledge of its substrate preferences was extended to different-sized PHB granules. It is shown that *Lt*PHBase is highly resistant to unfolding, with barriers typical for thermophilic enzymes, and shows a preference for low-molecular-mass PHB granules. These insights have implications for the long-term potential of *Lt*PHBase as an industrial PHB hydrolase and shed light on the evolutionary role that this enzyme plays in bacterial metabolism.

## Abstract 2

A future involving plastics that can be degraded and resynthesized requires an appropriate plastic to be found, plus green solutions that are compatible with waste-management systems. Here, the structure, stability and substrate-size preference are reported for a recently identified thermophilic serine hydrolase that degrades bioplastics such as polyhydroxybutyrate and other polymers.

## 1. Introduction

Pollution from plastics is a global problem that jeopardizes the health of our biosphere, with massive amounts of plastic deposited annually into landfills, freshwater sources and the ocean. Only 21% of petroleum-derived plastic waste is successfully managed through recycling and other waste-reducing processes. Most plastics have a lengthy half-life and take decades or longer to degrade. In addition, petroleum-based plastics can decompose into microplastics (<5 µm), which can re-enter human metabolism via the consumption of livestock, marine life and drinking water, all of which pose health hazards (Idris *et al.*, 2023 ▸; Andrady, 2011 ▸). Moreover, the SARS-CoV-2 pandemic has exacerbated the use of single-use disposable plastic materials in the form of personal protective equipment such as gloves (Idris *et al.*, 2023 ▸, Kaushal *et al.*, 2021 ▸).

Most synthetic plastics in use are petroleum-based (Mohanan *et al.*, 2020 ▸). Although efforts are under way to more efficiently degrade such plastics, an alternative is the use of bioplastics to manufacture consumable goods. Bioplastics are made from biomass and other readily available materials that are non-petroleum-based and that are naturally sourced (Tokiwa *et al.*, 2009 ▸). Common examples include polylactic acid (PLA) and poly(butylene adipate-co-terephthalate). These plastics are used in compostable cutlery and consumable goods that are commercially available today. Bioplastics degrade more quickly than fossil-fuel-based plastics (Tokiwa *et al.*, 2009 ▸). However, bioplastics can still produce microplastic particles if not processed properly during recycling, and not all bioplastic materials are readily degraded (Pascoe Ortiz, 2023 ▸).

Efforts to discover bioplastic-degrading organisms and adapt them to industrial waste-management schemes have significantly accelerated in recent years. Many microorganisms, including fungi, algae, bacteria and insects, possess bioplastic-depolymerizing enzymes (Yang *et al.*, 2020 ▸; Ping *et al.*, 2017 ▸; Sánchez, 2020 ▸). These organisms can scavenge for bioplastics, such as PLA or PHB, as an energy source. Biodegradation is an ideal solution for remediating bioplastic waste as it does not require the high temperatures necessary for thermal degradation and does not introduce additional pollutants into the environment.

Recently, we identified a PHBase from the thermophilic soil bacterium *Lihuaxuella thermophila* (*Lt*PHBase; UniProt A0A1H8IKU3). *Lt*PHBase exhibits optimal enzyme activity near 70°C (Thomas *et al.*, 2022 ▸). Like all PHBases, *Lt*PHBase contains the canonical Ser–His–Asp catalytic triad (Ser121–His270–Asp197). The crystal structure of *Lt*PHBase with serendipitously bound ethylene glycol (EG) and 2-propanol (IPA) revealed a relatively shallow active site compared with other PHBases (Thomas *et al.*, 2022 ▸), which we posited to explain its ability to accommodate many different substrates beyond PHB granules and films, such as PHB block copolymers and the unrelated polymers PLA and PCL.

Here, we present the crystal structure of *Lt*PHBase lacking any bound ligands and profile its chemical stability. We also extend our knowledge of substrate preferences to different-sized PHB granules. We show that *Lt*PHBase is highly resistant to unfolding, with barriers typical for thermophilic enzymes, and is an enzyme that prefers low-molecular-mass PHB granules. These insights have implications for the long-term potential of *Lt*PHBase for use as an industrial PHB hydrolase and shed light on the evolutionary role that this enzyme plays in bacterial metabolism.

## 2. Materials and methods

### 2.1. Chemicals and laboratory reagents

All chemicals and chromatography resins were purchased from Millipore–Sigma, St Louis, Missouri, USA, including all buffer components, media, isopropyl β-d-1-thiogalacto­pyranoside (IPTG), antibiotics and high-molecular-mass PHB polymer granules. Low-molecular-mass polyhydroxybutyrate polymer samples were obtained from Polysciences, Warrington, Pennsylvania, USA. The polymers were supplied as a powder/granulated powder and were used without further characterization. All laboratory supplies were purchased from Fisher Scientific. Chemically competent *Escherichia coli* cells were purchased from New England Biolabs, Beverly, Massachusetts, USA.

### 2.2. Expression, purification and activity assay

The enzyme was expressed and purified to homogeneity following the procedure described previously (Thomas *et al.*, 2022 ▸). The final protein preparation was concentrated to 10 mg ml^−1^ in 5.0 m*M* Tris–HCl pH 9.0 and frozen in small aliquots at −20°C until use. Enzymatic activity was determined via direct measurement of the β-hydroxybutyrate liberated during the depolymerization reaction using the MAK272 hydroxybutyrate assay kit from Millipore–Sigma, St Louis, Missouri, USA as described previously (Thomas *et al.*, 2022 ▸). The substrate concentration was 0.1–50 µ*M*, which spans the range of *K*_m_ values for each substrate.

### 2.3. Crystallization and structure determination

Crystallization conditions for apo *Lt*PHBase were identified by sparse-matrix screening that avoided 2-propanol (IPA) and ethylene glycol (EG). Crystals were grown at 25°C via hanging-drop crystallization in VDX plates with a reservoir buffer consisting of 18% PEG 4000, 100 m*M* Tris–HCl pH 9.0, 50 m*M* MgCl_2_, 50 m*M* KCl. A 4 µl drop volume was utilized by mixing 2 µl protein stock and 2 µl reservoir buffer. Hexagonal rod-shaped crystals formed in 3–4 days with approximate dimensions of 1–2 mm in length and a cross-sectional diameter of 0.2–0.3 mm. Crystals were harvested, incubated briefly in reservoir buffer supplemented with 20% PEG 400 and flash-cooled in liquid nitrogen. Data were collected at 100 K on beamline 5.0.1 at the Advanced Light Source (ALS) using an oscillation angle of 0.25° and a total rotation of 180°. The diffraction data were processed using *XDS* and scaled using *XSCALE* (Kabsch, 2010 ▸) using CC_1/2_ > 0.9 as a cutoff for resolution. The apo structure of *Lt*PHBase was solved by molecular replacement using PDB entry 8daj (Thomas *et al.*, 2022 ▸) as a search model. *Phenix.xtriage* did not detect any evidence of twinning (Liebschner *et al.*, 2019 ▸). The apo *Lt*PHBase structure was built in *Coot* (Emsley *et al.*, 2010 ▸) and refined using *phenix.refine* (Liebschner *et al.*, 2019 ▸; McCoy *et al.*, 2007 ▸; Table 1 ▸). *PDBePISA* (Krissinel & Henrick, 2007 ▸) was used to identify crystal contacts and *PyMOL* (Schrödinger) was used to generate figures. The structure has been deposited in the PDB with accession code 9byu.

### 2.4. Substrate docking

Apo *Lt*PHBase was submitted as the receptor and the hydroxybutyrate trimer from PDB entry 2d81 as the ligand to the online *EDock* server (Zhang *et al.*, 2020 ▸). The image for the top docked pose was generated in *PyMOL*.

### 2.5. Thermodynamic unfolding

*Lt*PHBase was chemically denatured in guanidinium hydrochloride (GdHCl; Millipore–Sigma, St Louis, Missouri, USA) at a concentration of 10 m*M* in 10 m*M* Tris–HCl pH 9.0. After mixing the protein with various amounts of the denaturant, the tubes were incubated at 25°C for 12 h to ensure that the samples were at equilibrium. Fluorescence emission intensity was measured using a Chirascan V100 spectropolarimeter (Applied Photophysics, Beverly, Massachusetts, USA) that was fitted with a CCD-array fluorescence detector, which simultaneously collects fluorescence emission intensity from 190 to 900 nm. The excitation wavelength of 280 nm was delivered via a fiber-optic cable connected to a light source. Emission spectra were analyzed in the region 300–450 nm. The fraction of unfolded protein was determined by fitting the fluorescence emission intensity to the relationship of Clarke & Fersht (1993 ▸),
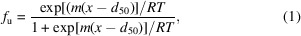
where *f*_u_ is the fraction of unfolded enzyme, *m* is the *m* value for cooperativity of the transition, *x* is the concentration of denaturant, *d*_50_ is the denaturation midpoint, *R* is the universal gas constant and *K* is the absolute temperature. The equilibrium constant *K*_u_ and the free-energy change Δ*G*_u_ for the unfolding reaction were calculated according to
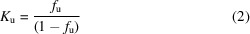
and
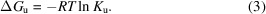
The free energy in the absence of denaturant, 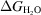, was calculated by a linear least-squares fit of the data (using *QtiPlot*; https://qtiplot.com/) in the linear portion of the unfolding curve to
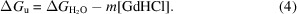


### 2.6. Kinetics of unfolding

The kinetics of the unfolding/refolding reaction as a function of denaturant concentration were determined using an SF3 stopped-flow device coupled to the Chirascan V100 spectropolarimeter. For unfolding reactions, *Lt*PHBase in 10 m*M* Tris–HCl pH 9.0 was rapidly mixed with GdHCl at concentrations ranging from 0.5 to 8 *M*. The dead time for mixing in the SF3 is approximately 2 ms. Single- or double-exponential functions were fitted to the experimental data using both the supplied instrument software and the *QtiPlot* scientific graphics package (https://qtiplot.com/). A single exponential provided the best fit, as evaluated by the coefficient of determination (*R*^2^), the root-mean-squared error (RMSE) and χ^2^ per degree of freedom (χ^2^/DOF) for multiple tested fits. The chevron plot was analyzed by determining the slope of the line between 0.5 and 3.5 *M* GdHCl to obtain the value of *m*_f_ and between 3.5 and 7.0 *M* GdHCl to obtain the value of *m*_u_. These slopes describe the dependence of the folding and unfolding reactions on GdHCl. Initially, the values of *k*_f_ and *k*_u_ in the absence of GdHCl, *i.e.* water only, were obtained by extrapolating the linear portions of the chevron curve to the *y* axis. Unfolding and refolding terms were also calculated by fitting the equation (Garg *et al.*, 2022 ▸)
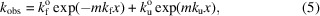
where *k*_obs_ is the observed rate constant, 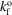 and 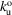 are the folding and unfolding rate constants in water, *m*_f_ and *m*_u_ are the slopes of the chevron curve on either side of the minimum and *x* is the concentration of GdHCl.

The value of *C*_m_, the GdHCl concentration at which 50% of the protein is unfolded, was determined as the GdHCl value at the minimum of the chevron curve and by calculating
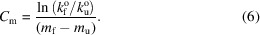
The free energy of the folding/unfolding reaction is calculated by
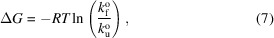
where Δ*G* is the free energy and 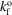 and 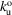 are the folding and unfolding rate constants in water.

## 3. Results

### 3.1. Crystal structure of apo *Lt*PHBase

In our previous *Lt*PHBase structure (PDB entry 8daj; Thomas *et al.*, 2022 ▸), two IPA molecules from the crystallization reservoir buffer and a molecule of EG from the cryoprotectant were bound serendipitously within the active site. These molecules, and a network of water molecules, formed a hydrogen-bonding network with surface loops and the catalytic triad. To test the hypothesis that *Lt*PHBase might adopt different active-site loop conformations in the absence of bound ligands, we identified new crystallization conditions that do not include IPA and we used PEG 400 as the cryoprotectant instead of EG.

**Figure 1:**
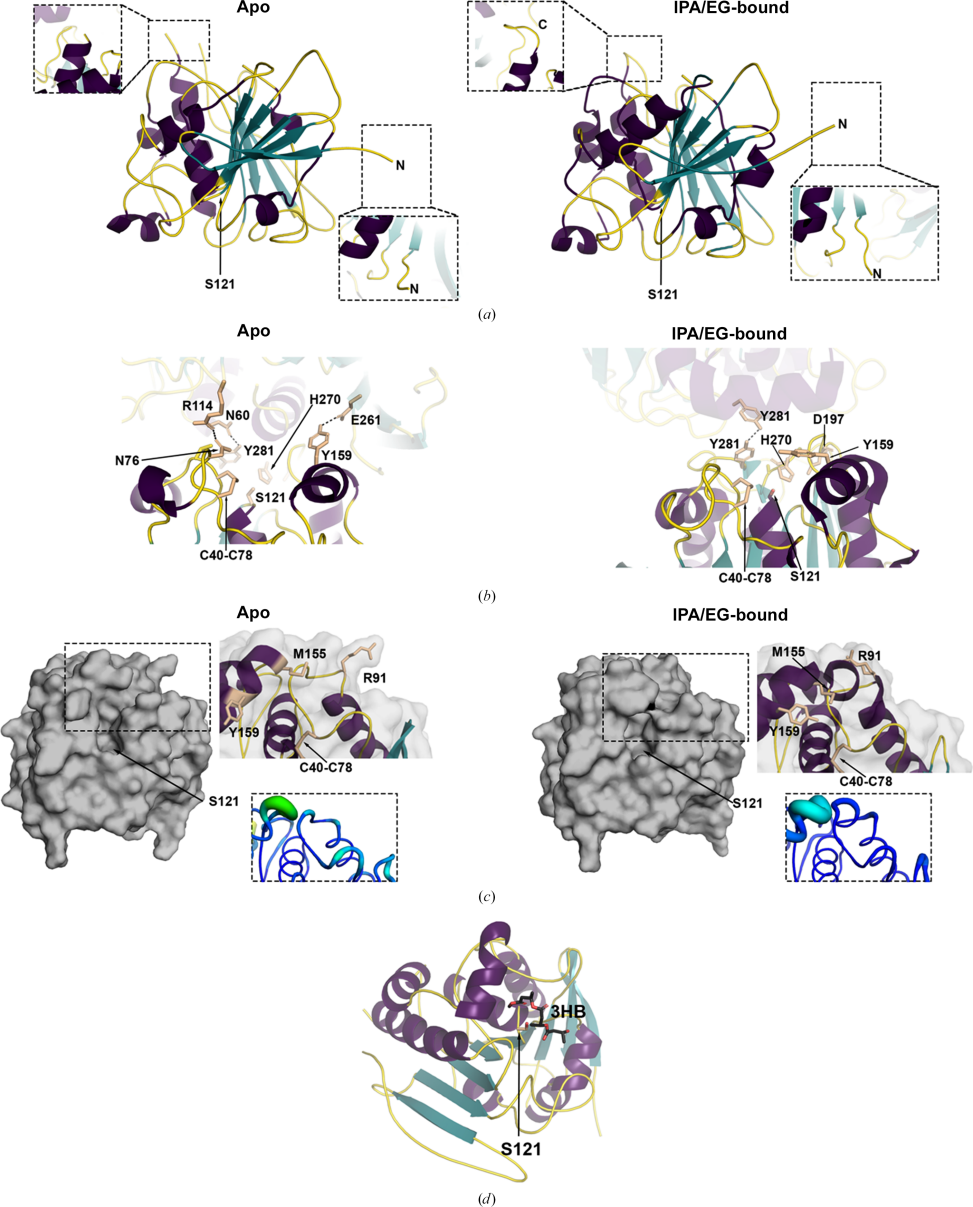
Structure of apo *Lt*PHBase. (*a*) Comparison of apo and IPA/EG-bound *Lt*PHBase, showing regions remote from the active sites that differ between the two structures. (*b*) Crystal contacts stabilizing the observed conformations of active-site loops. (*c*) Surface representation and zoom into differences in the active-site loops between apo and IPA/EG-bound *Lt*PHBase. The inset region is shown with a putty-style *B*-factor representation; the *B*-factor rainbow range is from 7 Å^2^ (blue) to 71 Å^2^ (red). (*d*) Docking model for the HB trimer.

The 1.75 Å resolution apo *Lt*PHBase structure was solved by molecular replacement using the IPA/EG-bound *Lt*PHBase structure (Table 1 ▸). The overall root-mean-squared deviation (r.m.s.d.) is 0.2 Å, indicating a high level of agreement between the two structures. The two most notable differences are distal from the active site. Firstly, the loop comprising residues 221–225 is disordered in apo *Lt*PHBase, whereas in the IPA/EG-bound structure the loop was readily modeled (Fig. 1 ▸*a*). Secondly, the five N-terminal residues adopt different conformations. These changes in the structure can be attributed to differences in crystal contacts (Figs. 1 ▸*a* and 1 ▸*b*) and suggest flexibility in these regions.

Subtle changes are also seen in the active site. Crystal contacts stabilize the active-site conformation observed in each structure (Fig. 1 ▸*b*). The original IPA/EG-bound structure is somewhat more occluded compared with the new apo structure in the region above Cys40 and Cys78, which appear to form a disulfide bond (Fig. 1 ▸*b*). Slight changes in the surface helix (residues 155–164) appear to be primarily responsible for this difference. Most notably, in the apo structure Met155 is oriented towards Phe81 and away from the Tyr159 side chain, which itself is in a new orientation compared with IPA/EG-bound *Lt*PHBase (Fig. 1 ▸*b*). Arg91 is also found in an alternative conformation compared with that in IPA/EG-bound *Lt*PHBase (Fig. 1 ▸*c*). In both structures the thermal *B* factors of Met155, Thr156 and Ser157 are considerably higher than the remaining helix and active-site environments (Fig. 1 ▸*c*), suggesting that this region is somewhat mobile and that additional loop conformations might be accessible in solution. Despite these differences, just like the IPA/EG-bound structure, a hydroxybutyrate trimer can still be docked computationally into the active site, indicating that the observed conformation is competent for catalysis (Fig. 1 ▸*d*). Taken together, the changes detected between the two structures imply some dynamics of surface features in solution, but the structures are otherwise nearly indistinguishable.

### 3.2. Thermodynamics and kinetics of *Lt*PHBase unfolding

**Figure 2:**
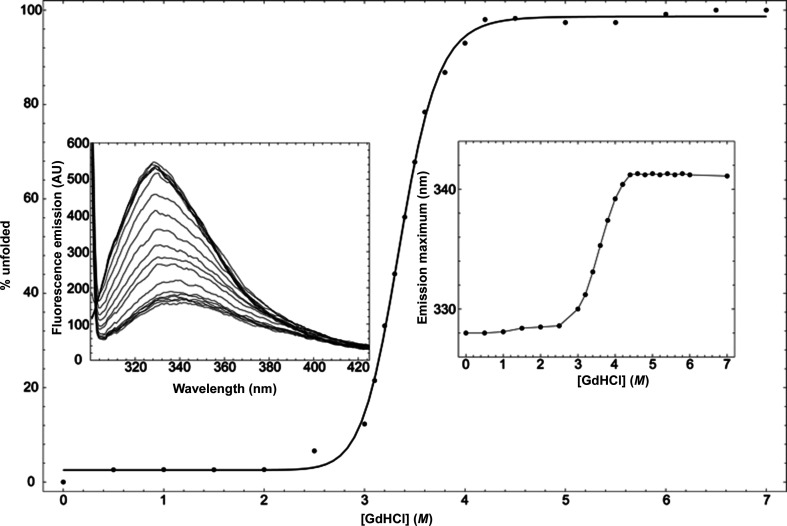
Equilibrium unfolding of *Lt*PHBase in GdHCl. The black circles represent the average percentage of unfolded protein determined by three independent experiments. The line is a fit of the data to a two-state unfolding mechanism. The left inset shows the fluorescence emission intensity spectrum for the concentration points measured (0.0 *M* GdHCl for the top-most curve to 7.0 *M* GdHCl for the bottom-most curve). The insert on the right shows the shift of the maximum fluorescence emission wavelength as a function of denaturant concentration.

To further characterize apo *Lt*PHBase, we next probed its room-temperature unfolding properties. Excitation at λ_ex_ = 280 nm monitors the microenvironment of the five buried tryptophans in *Lt*PHBase. The fluorescence emission intensity decreases, and the emission λ maximum is red-shifted from 328 to 341 nm, as a function of denaturant concentration. The unfolding transition begins in ∼2.8 *M* denaturant and is complete in 4.2 *M* GdHCl, with a midpoint of 3.4 *M* GdHCl. The unfolding reaction is best-fitted to a simple two-state model (N↔D; Fig. 2 ▸, Table 2 ▸). After the sample in 7.0 *M* GdHCl was dialyzed overnight versus 10 m*M* Tris–HCl pH 9.0 at 4°C, the enzyme regained activity, with a *K*_m_ of 8.3 m*M* and a *k*_cat_ of 3.2 s^−1^ when measured using the assay described in Section 2. These values are comparable to those for the native enzyme against the same substrate, indicating that chemical unfolding is fully reversible. The model fit shows that the enzyme stability in the absence of denaturant, 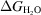, is 8.9 kcal mol^−1^.

**Figure 3:**
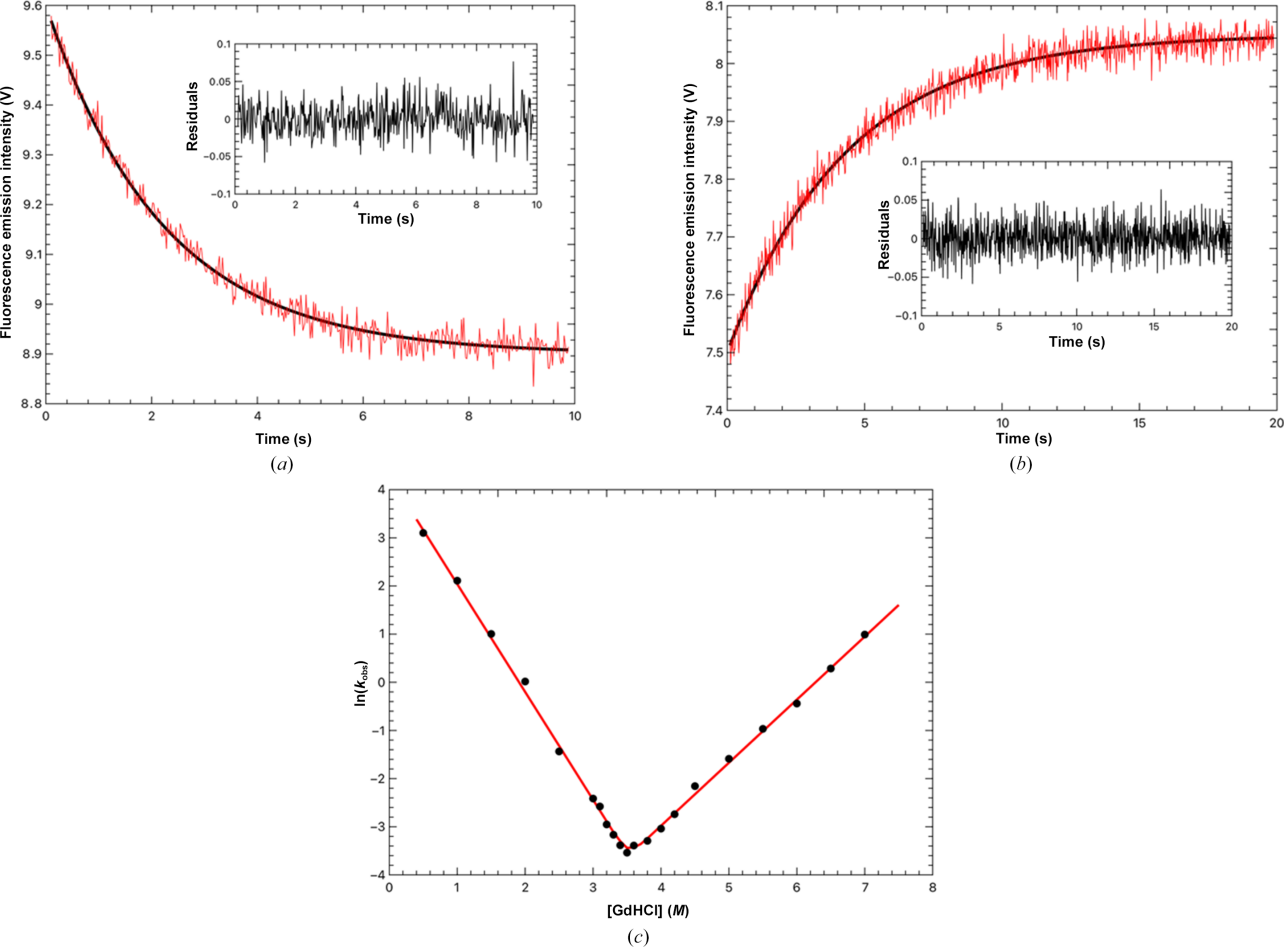
Kinetic unfolding and refolding of *Lt*PHBase. (*a*) Fluorescence-monitored refolding of *Lt*PHBase after the rapid mixing of the enzyme in 7.0 *M* GdHCl/reaction buffer with a tenfold excess of reaction buffer. (*b*) The gray lines in (*a*) and (*b*) are the fit to the kinetic data (in black). Each experimental curve is the average of 50 separate traces. The inset to both curves shows a single exponential fit of the kinetic data. The residuals are based on voltage differences between the fitted and observed data. (*c*) The GdHCl dependence of the natural logarithm of the observed refolding and unfolding rate constants. Each experimental point was determined as the average *k*_obs_ calculated from 50 independent kinetic traces. The fit of the experimental data to equation (5) is represented by a gray line.

Kinetic unfolding measurements further support a two-state unfolding transition for *Lt*PHBase at room temperature. Single mixing experiments of *Lt*PHBase and GdHCl indicated that *Lt*PHBase could rapidly be denatured at GdHCl concentrations above the midpoint of unfolding determined by equilibrium unfolding measurements (not shown). Fluorescence emission intensity at 328 nm (excitation wavelength of 280 nm) was monitored over the course of the unfolding or refolding reaction. For the unfolding reactions, enzyme at a concentration of 10 µ*M* in 10 m*M* Tris–HCl pH 9.0 was rapidly mixed with a 10× volume of GdHCl at concentrations between 0.5 and 8.0 *M*. When the folded enzyme is mixed with 8.0 *M* denaturant, rapid loss of fluorescence emission intensity at 328 nm is observed over 10 s. The curve is best-fitted to a single exponential, yielding a first-order rate constant for unfolding of 2.7 s^−1^ (Fig. 3 ▸*a*). Similarly, *Lt*PHBase refolding is observed in a rapid mixing experiment by diluting unfolded *Lt*PHBase in reaction buffer/7.0 *M* GdHCl with a tenfold excess of reaction buffer alone (final concentration of 0.7 *M* GdHCl), seen as a rapid increase in the fluorescence emission intensity over a 20 s time course. These kinetic data were also best-fitted to a single exponential, yielding a first-order rate constant for folding of 17.9 s^−1^ (Fig. 3 ▸*b*). The chevron plot (the logarithm of the observed unfolding/folding rate constant versus the denaturant concentration; Fig. 3 ▸*c*, Table 2 ▸) shows a linear dependence of *k*_obs_ on both sides of the GdHCl midpoint. Comparison of the slopes of the folding reaction, *m*_f_ (the left arm of the plot), and the unfolding reaction, *m*_u_ (the right arm of the plot), indicates that the folding reaction is faster than the unfolding reaction. Compared with the average folding and unfolding rates of thermophilic proteins that unfold via a two-state mechanism (Glyakina & Galzitskaya, 2020 ▸), *Lt*PHBase folds at a similar rate [ln(*k*_f_) = 4.22 compared with an average ln(*k*_f_) of 4.75 ± 1.2] but unfolds faster [ln(*k*_u_) = −8.55, average ln(*k*_u_) = −5.63 ± 1.12; range −12 to 4].

In summary, both kinetics and equilibrium experiments indicate that *Lt*PHBase unfolding at room temperature is best described as a simple two-state process. The denaturant midpoint and free-energy values are within 1.1 kcal mol^−1^. This, and the symmetric shape of the chevron plot, indicates that there is not likely to be an intermediate form in the unfolding/refolding pathways and that the reaction is fully reversible.

### 3.3. Enzymatic activity versus PHB molecular mass

Given the shallow active site and broad substrate repertoire, we next probed the effect of PHB polymer size on the activity of *Lt*PHBase. Enzyme kinetic data were acquired on PHB samples with molecular masses ranging from 500 to 350 000 Da (Table 3 ▸). *Lt*PHBase hydrolyzes all substrates within this molecular-mass range; however, there is an inverse relationship between PHB mass and the catalytic parameters. Namely, the Michaelis constant, *K*_m_, which is indicative of substrate binding, ranges from an average of 3.5 to 19.2 m*M* and the catalytic efficiency, *k*_cat_, ranges from 11.2 to 0.82 s^−1^ over the PHB mass range from 500 to 350 000 Da. In sum, *Lt*PHBase prefers lower-molecular-mass PHB. Whether the *Lt*PHBase reaction is endohydrolytic or exohydrolytic is unknown, but the enzyme rates could be higher for the lower-molecular-mass PHBs if the catalytic mechanism is solely endohydrolytic.

## 4. Discussion

A future that uses an environmentally circular bioplastics scheme would likely involve bioplastic degradation as part of industrial waste management. Such a process would further require a bioplastic-degrading enzyme that is able to function at and withstand the associated harsh conditions. To this end, we identified a promiscuous PHBase from the thermophilic soil bacterium *L. thermophila* (*Lt*PHBase)*. Lt*PHBase can accommodate many different substrates (Thomas *et al.*, 2022 ▸).

Despite the fact that other PHBase structures have active sites with a range of accessibilities (Hisano *et al.*, 2006 ▸; Jendrossek *et al.*, 2013 ▸), our study shows that the active site of *Lt*PHBase has loops that adopt virtually the same position whether in the apo form or bound by IPA/EG. Thus, it appears that PHBases have diverged evolutionarily with regard to the degree of conformational change that is required to accommodate PHB substrate(s) into the binding cleft and ultimately into position by the catalytic triad. This mechanistic difference is most likely to drive the observed substrate preferences.

To our knowledge, *Lt*PHBase is the first PHBase to be surveyed for PHB molecular-mass preference. Given that *Lt*PHBase hydrolyzes multiple different substrates, we hypothesized that there would not be a substantial molecular-mass preference. Our experiments did not support this hypothesis. *Lt*PHBase is likely to prefer lower-molecular-mass, non-film polymers due to the apparent lack of conformational access to the catalytic triad, as seen in our apo structure. The lower-molecular-weight polymer samples are more readily accommodated into the binding cleft of the enzyme, so that the labile ester bond is positioned within the catalytic triad. The 350 000 Da sample is a poorer substrate than the PHB film initially utilized to characterize the enzyme (Thomas *et al.*, 2022 ▸). Yet, the enzyme is capable of hydrolyzing high-molecular-mass PHB polymers and films, so there is potentially a change in the active-site loop conformation that allows these larger substrates to be accommodated in the active site that is not captured in the current crystal structure. The importance of the active-site loops in *Lt*PHBase are underscored by unsuccessful attempts to express a soluble enzyme with a shortened loop in this region (not shown).

Generally, thermophilic enzymes are kinetically slower than their mesophilic or psychrophilic counterparts at their optimum temperature due to the need of thermophiles to invest more structural reorganization energy during the electrostatic reorganization process (Roca *et al.*, 2007 ▸). Direct kinetic comparisons across known PHBases are hampered by differences in the available PHB substrates, primarily molecular mass, average granule diameter or film dimensions, used in different laboratories for the enzymatic assays. Still, casual inspection of the rates for mesophilic forms of the enzyme [for example PHBases from *Aeromonas caviae* (Amir *et al.*, 2024 ▸), *Microbacterium paraoxydans* (Sayyed *et al.*, 2019 ▸) and *Penicillium expansum* (Gowda & Shivakumar, 2015 ▸)] indicate that *Lt*PHBase is slower than these mesophilic PHBases. Thus, another curiosity regarding *Lt*PHBase is the interplay between its slow kinetics and the aforementioned substrate preferences. One potential caveat of the current work is that crystallization was performed and the kinetics of folding and unfolding were determined at room temperature, which is ∼30°C below the optimal temperature of the enzyme kinetics. Although this may affect the absolute values of rate constants and loop positions in the crystal structure, the results are directionally correct because the enzyme is functional at room temperature. Future efforts will be directed towards solution dynamics of *Lt*PHBase not accessible in the crystalline state or kinetics at room temperature, which may in turn underlie preferences for both different sizes and types of substrates.

The high degree of thermostability of *Lt*PHBase reported by us previously, as expected, is manifested in a high degree of resistance to chemical denaturation, with an apparent transition midpoint of 3.4 *M* GdHCl. In general, resistance to denaturation is typical of thermophilic and hyperthermophilic enzymes (Feller, 2018 ▸). For example, the MTH1880 protein from *Methanobacterium thermoautotrophicum* has a chemical unfolding midpoint of 4.0 *M* GdHCl (Kim *et al.*, 2016 ▸). Stabilization of the native structure is a hallmark of thermostable enzymes and a mechanism that is entropically driven (Pica & Graziano, 2016 ▸). Although there is not a singular mechanism that explains thermophilic enzymes, resistance to unfolding is often associated with more well packed hydrophobic cores and an increase in the number of electrostatic interactions relative to mesophilic counterparts (Reed *et al.*, 2013 ▸). *Lt*PHBase has an extensive hydrophobic core and a substantial number of electrostatic interactions. Kinetic stability is in part afforded by a slower unfolding rate and a faster folding rate (Mukaiyama & Takano, 2009 ▸), and in the case of *Lt*PHBase the folding rate is faster than its chemical unfolding rate. Interestingly, this is a smaller difference in rates than has been seen in other thermophilic enzymes, but still results in an increase in the activation barrier to unfolding (Karshikoff *et al.*, 2015 ▸; Wittung-Stafshede, 2004 ▸).

*Lt*PHBase belongs to the secreted subfamily of PHBases, which most likely function to scavenge hydroxybutyrate (HB) from a few environmental biopolyester polymers. Presumably, the liberated HB is transported into the bacterium for use as an energy source. However, there are no other PHB-cycle enzymes that can be identified in the annotated genome or by *BLAST* analyses (Altschul *et al.*, 1990 ▸) against the *L. thermophila* genome using homologous PHB-biosynthesis enzymes (not shown). *L. thermophila* is not unique in this regard. A *BLAST* search of *Lt*PHBase against microbial genomes reveals PHBase homologs in organisms such as *Bacillus aquiflavi* and *Shouchella shacheensis* that similarly lack PHB-biosynthesis enzymes, although these are mesophiles. In the long term, beyond clarification of the relationship between the substrate scope and kinetics of *Lt*PHBase for the potential use of PHB as a renewable biopolymer, is the solution of the mystery of why *L. thermophila* and other organisms have evolved to utilize an imported polymer as an energy source.

## Supplementary Material

PDB reference: apo *Lt*PHBase, 9byu

## Figures and Tables

**Table 1:** Data-collection and refinement statistics Values in parentheses are for the highest resolution shell.

Wavelength (Å)	1
Resolution range (Å)	76.72–1.75 (1.81–1.75)
Space group	*P*6_4_
*a*, *b*, *c* (Å)	88.58, 88.58, 49.81
Total reflections	145491 (1173)
Unique reflections	20114 (819)
Multiplicity	7.2 (1.4)
Completeness (%)	89.0 (36.8)
Mean *I*/σ(*I*)	18.03 (1.2)
Wilson *B* factor (Å^2^)	16.75
*R*_merge_	0.0656 (0.113)
*R*_meas_	0.0696 (0.153)
*R*_p.i.m._	0.0228 (0.102)
CC_1/2_	0.998 (0.964)
Reflections used in refinement	20114 (819)
Reflections used for *R*_free_	1985 (80)
*R*_work_	0.143 (0.265)
*R*_free_	0.191 (0.360)
No. of non-H atoms	
Total	2585
Macromolecules	2325
Ligands	0
Solvent	260
Protein residues	300
R.m.s.d., bond lengths (Å)	0.008
R.m.s.d., angles (°)	0.87
Ramachandran favored (%)	98.3
Ramachandran allowed (%)	1.7
Ramachandran outliers (%)	0.00
Rotamer outliers (%)	0.82
Clashscore	3.99
Average *B* factor (Å^2^)	
Overall	18.42
Macromolecules	17.50
Solvent	26.68

**Table 2:** Thermodynamic and kinetic chemical denaturation values for *Lt*PHBase

Thermodynamic determination	
[GdHCl]_1/2_ (*M*)	3.41
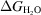 (kcal mol^−1^)	8.9
*m* (kcal mol^−1^ *M*^−1^)	2.72
Kinetic determination	
*C*_m_ (*M*)	3.49
*m*_u_	1.35
*m*_f_	−2.20
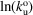	−8.55
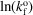	4.22
Δ*G* (kcal mol^−1^)	7.8

**Table 3:** Kinetic constants for purified *L. thermophila* PHBase versus polymer molecular mass

PHB molecular mass (Da)	*K*_m_† (µ*M*)	*k*_cat_† (s^−1^)	*k*_cat_/*K*_m_‡ (s^−1^ *M*^−1^) × 10^−6^
500	3.5 (0.2)	11.2 (0.1)	3.2 (0.2)
1000	4.8 (0.1)	7.1 (0.1)	1.5 (0.04)
2000	5.0 (0.2)	6.3 (0.1)	1.3 (0.05)
3000	6.3 (0.2)	5.4 (0.2)	0.86 (0.04)
5000	7.4 (0.2)	4.1 (0.1)	0.55 (0.02)
10000	8.6 (0.1)	3.0 (0.1)	0.35 (0.01)
50000	12.0 (0.2)	2.1 (0.1)	0.18 (0.01)
350000	19.2 (0.1)	0.82 (0.2)	0.043 (0.01)

† For *K*_m_ and *k*_cat_, the values are the mean of three independent experiments and the values in parentheses are the standard deviation.

‡ For *k*_cat_/*K*_m_, the values in parentheses are the calculated Gaussian error propagation: Δ*Z* = *Z*[(Δ*X*/*X*)^2^ + (Δ*Y*/*Y*)^2^]^1/2^, where *X* is *k*_cat_ and *Y* is *K*_m_.
